# Geo-social gradients in predicted COVID-19 prevalence in Great Britain: results from 1 960 242 users of the COVID-19 Symptoms Study app

**DOI:** 10.1136/thoraxjnl-2020-215119

**Published:** 2020-12-29

**Authors:** Ruth C E Bowyer, Thomas Varsavsky, Ellen J Thompson, Carole H Sudre, Benjamin A K Murray, Maxim B Freidin, Darioush Yarand, Sajaysurya Ganesh, Joan Capdevila, Elco Bakker, M Jorge Cardoso, Richard Davies, Jonathan Wolf, Tim D Spector, Sebastien Ourselin, Claire J Steves, Cristina Menni

**Affiliations:** 1 Twin Research, King's College London, London, UK; 2 School of Biomedical Engineering & Imaging Sciences, King's College London, London, UK; 3 MRC Unit for Lifelong Health and Ageing, University College London, London, UK; 4 Zoe Global Limited, London, UK

**Keywords:** clinical epidemiology, infection control

## Abstract

Understanding the geographical distribution of COVID-19 through the general population is key to the provision of adequate healthcare services. Using self-reported data from 1 960 242 unique users in Great Britain (GB) of the *COVID-19 Symptom Study app,* we estimated that, concurrent to the GB government sanctioning lockdown, COVID-19 was distributed across GB, with evidence of ‘urban hotspots’. We found a geo-social gradient associated with predicted disease prevalence suggesting urban areas and areas of higher deprivation are most affected. Our results demonstrate use of self-reported symptoms data to provide focus on geographical areas with identified risk factors.

The COVID-19 epidemic has led to large-scale closures and lockdown measures worldwide with the British government sanctioning lockdown from 23 March 2020 (https://www.gov.uk/government/speeches/pm-address-to-the-nation-on-coronavirus-23-march-2020).

Early in the pandemic, case distribution was not evenly spread across countries, with dense urban centres being the most affected.^1^ Individuals in deprived areas have lower life expectancy,^2^ are more likely to have multiple underlying comorbidities, have a higher level of influenza-associated hospitalisation^3^ and therefore could be more susceptible to COVID-19.^2^

Based on the known socioeconomic health gradient, we hypothesised that individuals in deprived areas were at greater risk of contracting COVID-19. Understanding the geographical distribution of the virus in a socioeconomic context is key to assist adequate healthcare resourcing, particularly intensive care beds.^4^

Here we investigated the geographical distribution of COVID-19 in Great Britain (GB) and its association with area-level deprivation using self-reported data from almost 2 million users of the *COVID-19 Symptom Study.* 5

We studied 1 960 242 unique GB app users (20–69 years old) reporting on COVID-19 symptoms, hospitalisation, reverse-transcription PCR (RT-PCR) test outcomes, demographic information and pre-existing medical conditions (online supplemental methods) over 23 days (29 March–19 April) of major social distancing measures (‘lockdown’). We computed a proxy of contracting COVID-19, based on reported symptoms^6^ (positive predicted value=0.69 (0.66; 0.71) (online supplemental methods). We then calculated a predicted prevalence as the proportion of app users that we predicted to have COVID-19 within each area (online supplementary figure S1).

10.1136/thoraxjnl-2020-215119.supp1 Supplementary data

10.1136/thoraxjnl-2020-215119.supp2 Supplementary data

Following aggregation of variables to local authority district level (LAD/geographic unit representing ~17 000 individuals), we tested the geographical distribution of predicted prevalence at eight different time points spanning 23 days. We used Local Moran’s I tests, which assess for non-random spatial distribution and clustering of a feature and can be used to identify disease hotspots and cold spots relative to the mean GB predicted prevalence^7^ (online supplemental methods).

Next, we used data from the eight different time points and used multivariable mixed-effects models to investigate the association of predicted area-level prevalence (at middle super output area level (MSOA)) and deprivation (as captured by the Index of Multiple Deprivatio) adjusting for different factors including geo-social mediators and confounders (air pollution, general practitioners per MSOA, household density and urbanicity) area level aggregates of obesity and comorbidities) and area-level adjusted mean age and sex and spatial autocorrelations^8^ (online supplemental methods).

table table 1 1 and online supplemental table S1. The number of predicted COVID-19 positive individuals ranged between 15 991 and 79 378.

10.1136/thoraxjnl-2020-215119.supp4 Supplementary data

**Table 1:** Demographic characteristics of the study population at eight time points

	29 March 2020	1 April 2020	4 April 2020	7 April 2020	10 April 2020	13 April 2020	16 April 2020	19 April 2020	All unique users
N	1 324 843	1 431 515	1 142 923	1 083 601	995 157	985 860	980 608	1 164 262	1 960 242
Predicted COVID-19 (n/%)	60 827	79 378	62 508	48 418	30 132	22 352	16 586	15 991	117 614
	(4.6)	(5.6)	(5.5)	(4.5)	(3.0)	(2.3)	(1.7)	(1.4)	(6.0)
Average number of reports per user	2.9	3.8	4.2	4.7	5	5	5	4.5	4.4
Age, years (median (IQR))	41 (21)	41 (21)	43 (21)	44 (22)	45 (21)	45 (21)	46 (21)	45 (21)	42.2 (21.8)
Male, (n/%)	426 923	459 620	365 078	353 233	327 608	327 620	327 114	388 378	654 950
	(32.2)	(32.1)	(31.9)	(32.6)	(32.9)	(33.3)	(33.3)	(33.4)	(33.4)
Obesity, %	21.3	21.4	20.7	20.3	21.6	22.1	21.4	21.7	21.5
Kidney disease, %	0.5	0.5	0.5	0.5	0.5	0.6	0.6	0.6	0.5
Lung disease, %	12.2	12.3	12.5	12.5	12.4	12.4	12.4	12.4	12.2
Diabetes, %	2.4	2.5	2.7	2.7	2.8	2.9	2.9	2.9	2.4
Smokers, %	10.5	10.5	9.7	9.4	9.0	8.8	8.7	9.0	10.4
Heartdisease, %	1.4	1.4	1.6	1.6	1.7	1.7	1.7	1.7	1.4

Obesity: BMI >=30 kg/m^2^.

At each time point, we only include users who have made an assessment in the previous 7 days. Exclusion criteria are listed in the supplements. Users are asked daily whether (or not) they have any symptoms. Predicted COVID-19 was calculated on users who reported on symptoms. Users who reported having no symptoms were included in the area-level predicted prevalence estimates (please see the supplements for details).

BMI, body mass index.

Local Moran’s I showed that predicted COVID-19 prevalence clustered in urban areas across GB when considered as a proportion of the population per LAD^7^ (figure 1 and online supplemental figure S2) adjusting for multiple testing. Predicted prevalence decreased over time, consistent with ‘lockdown’ (figure 1 and online supplemental figure S2) (pairwise Wilcoxon rank-sum tests, prevalence: all time points except T2:T3 and T1:T4, p<0.001), but some hotspots remained.

10.1136/thoraxjnl-2020-215119.supp3 Supplementary data

**Figure 1:**
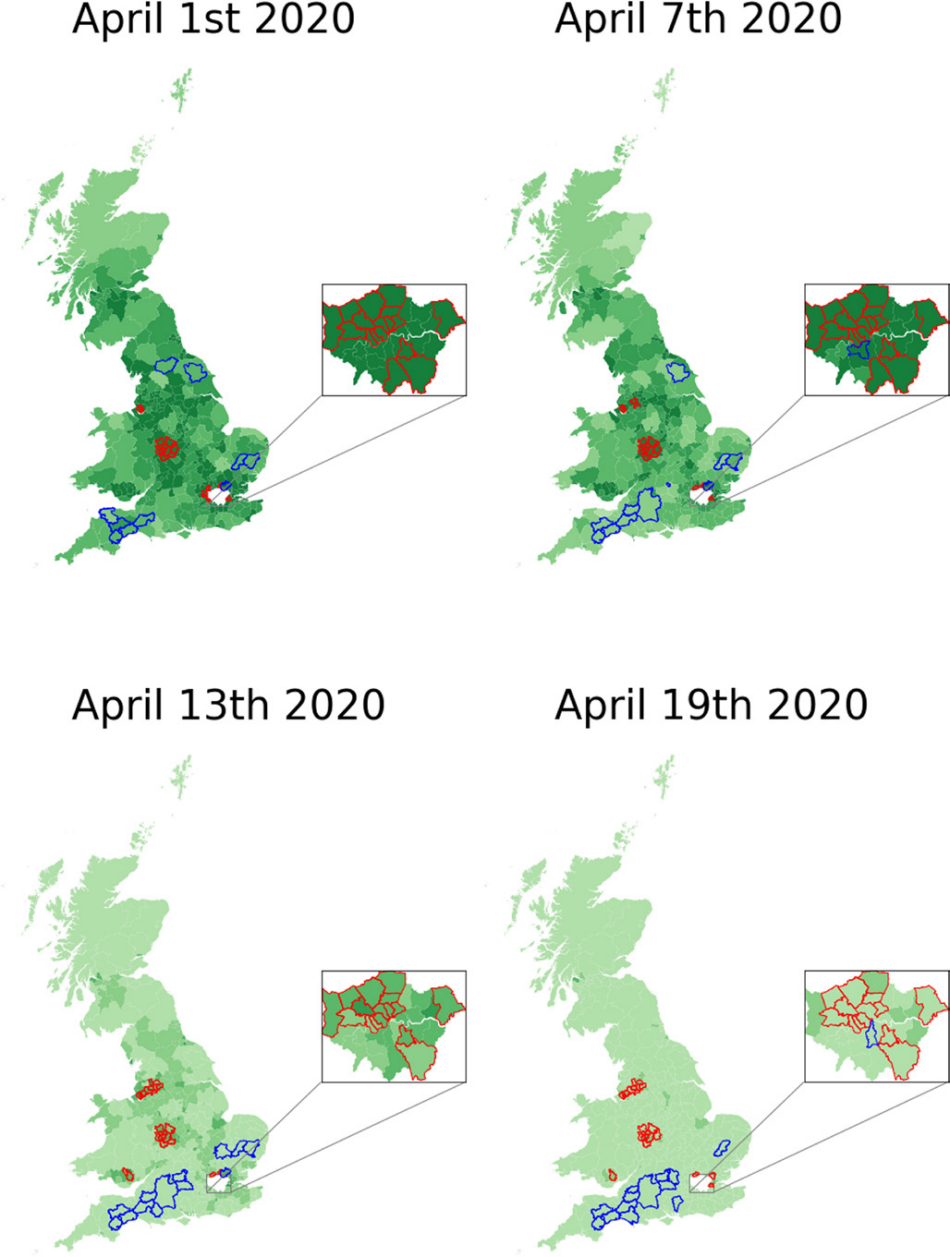
Geographical distribution of predicted COVID-19 prevalence across four time points. Prevalence is presented as proportional to the responders per local authority district (LAD). Analyses are adjusted for multiple testing using Benjamini- Hochberg false discovery rate correction (p<0.05). Inset highlights London where LAD areas are smaller. Hot and cold spots are defined relatively to their neighbours and the mean GB predicted prevalence. Red/blue coloured perimeter lines around each LAD denote hotspot/coldspot.

In the MSOA-level analysis, area-level deprivation was significantly associated with predicted area-level prevalence in all models (M1–M6, see online supplemental table S2), including in the full model (M6) when adjusting for all geo-social covariates and comorbidities (M6: Beta (95% CI)=−0.15 (−0.17 to –0.130, p<0.001). This suggests that people in deprived areas were at higher risk.

Predicted COVID-19 prevalence was higher in urban areas compared with rural and in more deprived areas compared with less deprived. This could reflect the likelihood of individuals in more deprived areas working/living with people whose vocations mean they are unable to work from home and are thus more likely to be exposed to circulating COVID-19. Accumulation of socioenvironmental exposures across the life course are known to contribute to a greater health deficit and disease burden^2^; our results suggest that COVID-19 is no exception.

Moreover, our study illustrates how app data could be used to successfully monitor COVID-19 over time and identify hotspots as the viral pandemic progresses and social distancing measures are implemented or eased. Using this method, we detected a geo-social gradient associated with prevalence in the context of COVID-19, suggesting the focus of resources should be on deprived urban areas.

Our study has some limitations and assumptions. We used self-reported data on symptoms that can lead to bias. For example, should users in deprived areas report more symptoms due to a facet of the socioeconomic environment (eg, higher air pollution), this could lead to an incorrectly higher predicted prevalence in deprived areas. Second, app users are a self-selected group, not representative of the general population. Our approach to adjust for age and sex differences at MSOA level is unlikely to sufficiently overcome selection and collider bias.^9^ Third, our predicted COVID-19 prevalence is not from confirmed tests via RT-PCR, but rather based on self-reported symptoms. Additionally, we assume that people who have symptoms or have been exposed to COVID-19 are equally likely to use the app as those who do not. We performed a sensitivity analysis by rerunning the pooled analysis on individuals who were self-reportedly healthy at sign up and found the observed associations remained (online supplemental table S3), suggesting selection bias associated with being unhealthy at sign up is not influencing the observed associations of COVID-19 and deprivation. We also assume that people report symptoms in the same way and that their drop-out patterns do not differ by space, time and symptom reports. Finally, we aggregated data at MSOA level that could lead to ecological bias. We also cannot conclude that deprivation increased COVID-19 prevalence, as there could be unmeasured confounders or other factors.

Future work should check our assumptions and seek to integrate these data with data on area-level morbidity, extended pollution data, ethnicity and disease severity. Indeed, higher mortality has been observed among minority ethnic groups,^10^ and disentangling the environmental and biological factors contributing to greater disease burden in both deprived areas and among ethnic minorities is an essential focus of future work to ensure resources and intervention are better assigned.
