# Supplementary material for: Geo-social gradients in predicted COVID-19 prevalence in Great Britain: results from 1 960 242 users of the COVID-19 Symptoms Study app
Source: Thorax. 2020 Dec 29;76(7):723–5. doi: 10.1136/thoraxjnl-2020-215119 (PMC8223682; doi:10.1136/thoraxjnl-2020-215119)
Supplement: Supplementary data [file thoraxjnl-2020-215119supp001.pdf]

Supplementary Methods

Study setting and participants

The COVID Symptom Study app developed by Zoe with scientific input from researchers and clinicians at King's College London and Massachusetts General Hospital, was launched in GB on Tuesday the 24th March 2020 (https://covid.joinzoe.com/) and in the 23 days (March 29th – April 19th) immediately after the UK lockdown (https://www.gov.uk/government/speeches/pm-statement-on-coronavirus-22-march-2020) was introduced, it reached 2,266,235 unique GB users, making 9,108,769 assessments (e.g. an average user is included in 4 out of 8 timepoints). Referrals/word of mouth, press and eventually partnerships with charities and the Welsh and Scottish governments drove usage.

The app enables capture of self-reported information related to COVID-19 infections. On first use, the app records self-reported location, age, and core health risk factors. With continued use, participants provide daily updates on symptoms, health care visits, COVID-19 testing results, and if they are self-quarantining or seeking health care, including the level of intervention and related outcomes. Individuals without apparent symptoms are also encouraged to use the app. Through direct updates, the research team can add or modify questions in real-time to capture new data to test emerging hypotheses about COVID-19 symptoms and treatments. Importantly, participants enrolled in ongoing epidemiologic studies, clinical cohorts, or clinical trials, can provide informed consent to link data collected through the app in a Health Insurance Portability and Accountability Act (HIPAA) and General Data Protection Regulation (GDPR)-compliant manner with extant study data they have previously provided or may provide in the future.

In this study, we included 1,960,242 unique users as outlined in the flow diagram below (**Figure A**). Briefly, out of 2,415,843 unique app uses who reported on the COVID-19 symptom Study App between 29th March 2020 and 19th April 2020, we excluded (i) 149,608 non GB users; (ii) 88,422 users who only reported on the earliest app-version that did not include loss of smell and taste (the strongest single predictor of COVID-19[1,2]); (iii) 66,975 reporting BMI outside the biological range; (iv) 148,111 users younger than 20 or older than 69; (v) 1007 with missing biological sex at birth or who were not assigned male or female as their biological sex at birth; (v) 1478 users who did not report on pre-existing medical conditions (**Figure A**).

Figure A. Flow diagram representing the study subjects' inclusion criteria.

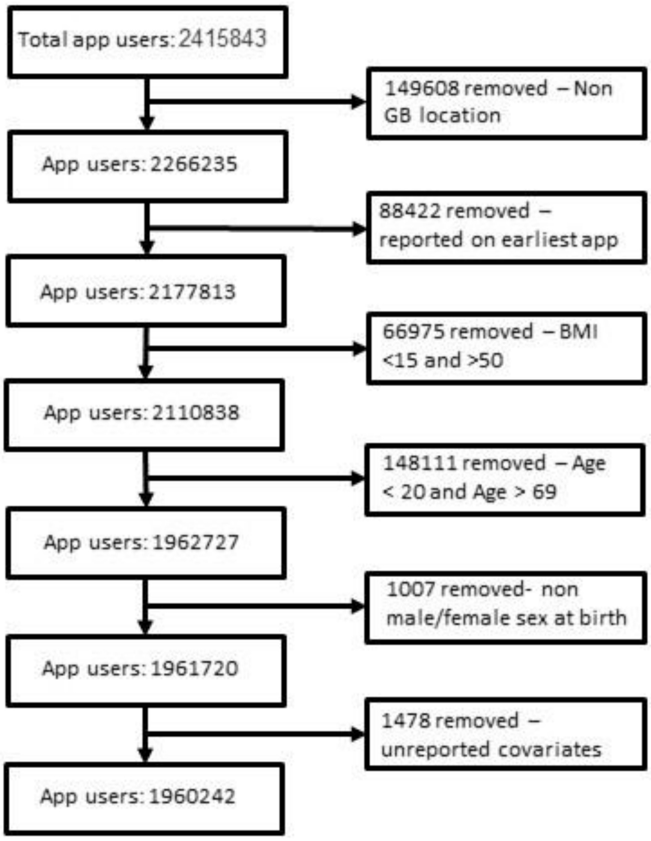

Geographic clustering of COVID-19 prevalence

Because we were primarily interested in understanding the geography of COVID-19 distribution, and how aspects of an area, in particular area-level deprivation, associated with COVID-19 prevalence we aggregated user data at different GB geographic areas. This was particularly of use as the geosocial variables considered (please see below) are also defined geographically and are time invariant (as they are not defined by the app users themselves but by GB geographic area).

The maps (**Figure 1, S2**) were created using a shapefile of Local Authority Districts (LADs) from the Office for National Statistics (ONS) using the geopandas package in Python. Overlaid on the map are statistically significant 'hot-spots' and 'cold-spots' at LAD level. To assess the significance of these regions, we used Local Moran's I test, as introduced below. In order to do this, spatial weights were

calculated to create a spatially lagged COVID-19 prevalence variable for each LAD. Because our geographical units share borders we assume a queen criterion, which assumes equal weights of neighbouring areas, which is appropriate for defining these. Islands were considered to have zero neighbours. We adjusted for multiple testing using the Benjamini & Hochberg method (‘p.adjust’) and used the ‘spdep’ package in R for the Local Moran’s and calculation of the spatial lag. This approach of calculating the spatial lag was repeated at the middle super output area level (MSOA) level (below).

Hotspot and Coldspot definition

Predicted prevalence hotspots at LAD levels were defined using Local Moran’ s I. The Moran’s I statistic gives a value indicating the spatial clustering of a variable relative to its neighbours. Where there are significant (false discovery rate (FDR)adjusted $p < 0.05$) high positive local Moran’s I in high value neighbourhood (i.e. where the significant area also had a predicted prevalence greater than the mean predicted prevalence and greater than the mean of the lagged variable, which effectively represents how similar COVID-19 prevalence is to the areas that surround it) this implies the area can be considered a ‘hotspot’[3]. This method ensures we do not consider areas as hotspots where they may have higher predicted prevalence to the surrounding areas but are lower than average for the UK, although it might miss areas that are surrounded on all borders by other areas which would be considered hotspots. A coldspot is assessed similarly using Local Moran’s I, but where the area is less than the mean and mean of the lagged variable.

Sources of geographic data

Index of Multiple Deprivation (IMD)

The IMD was downloaded from the relevant government websites as below, and the most recent IMD available at time of analysis was used:

- English (2019): https://www.gov.uk/government/statistics/english-indices-of-deprivation-2019
- Scottish (2016): https://www2.gov.scot/Topics/Statistics/SIMD
- Welsh (2019): https://statswales.gov.wales/Catalogue/Community-Safety-and-Social-Inclusion/Welsh-Index-of-Multiple-Deprivation/WIMD-2019

Because the IMD is calculated in each devolved administration using slightly different methodology, and because of the different number of areas in each country, ranks are not directly comparable.

Therefore, we used within-country defined deciles. As the IMD is calculated for smaller area geographies than MSOA, we calculated the average IMD per MSOA. This was then categorised into quintiles where 1 is the least deprived and 5 is the most deprived.

Rural-urban gradient (RUC)

The RUC was downloaded from the relevant government websites as below:

- England and Wales RUC (2011): https://data.gov.uk/dataset/9c0e093d-d267-4eb8-90d8-54475ab4d1ff/rural-urban-classification-2011-of-middle-layer-super-output-areas-in-england-and-wales
- Scotland RUC (8 fold classification): https://www2.gov.scot/Topics/Statistics/About/Methodology/UrbanRuralClassification

The resulting scale runs from 1 – 8, where 1 is the most urban and 8 is the least.

Nitrogen Oxide (NOx) data

We used NOx pollution data from the Department of Environment, Food and Rural Affairs (https://uk-air.defra.gov.uk/data/) for England, Scotland and Wales from 2018. Data is provided with Ordinance Survey 1km^2 grid resolution which was used to calculate per MSOA air pollution by taking the area-weighted average of the readings.

General Practitioners (GPs)/MSOA

GPs addresses were used to derive the number of GPs from each MSOA, from the following data sources:

- England & Wales:https://digital.nhs.uk/services/organisation-data-service/data-downloads/gp-and-gp-practice-related-data
- Scotland: https://www.opendata.nhs.scot/ne/dataset/general-practitioner-contact-details/resource/b092b69f-0838-408e-bb89-082562f0e1cd

Average household number

This figure was derived from data by dividing the number of houses with at least one usual occupant with the total population for the same area.

Data sources for occupancy data were downloaded from the following sources:

- England & Wales (table PHP01 2011): https://www.nrscotland.gov.uk/statistics-and-data/statistics/statistics-by-theme/households/household-estimates/small-area-statistics-on-households-and-dwellings
- Scotland: https://www.nrscotland.gov.uk/statistics-and-data/statistics/statistics-by-theme/households/household-estimates/small-area-statistics-on-households-and-dwellings

MSOA-level mixed-effects models

We employed multivariable mixed-effects models to understand the relationship of predicted COVID-19 prevalence at MSOA level with deprivation. As a reminder, these models were ran at MSOA-level rather than individual-level. This included the following variables:

The Index of Multiple Deprivation, our primary explanatory variable (IMD, categorised into quintiles generated on the average IMD within each MSOA, where 1 is most deprived and 5 is least, and considered as a continuous variable).

Other considered geosocial factors included a rural-urban gradient (RUC, considered as a continuous variable where 1 is the most urban and 8 is the most rural), General practitioners per population in MSOA (GPs/MSOA, where a higher number indicates more GPs per individual by MSOA), average household number (calculated as number of inhabited dwellings/MSOA population, where a higher number indicates a higher average number of individuals per household). Because it was on a very different scale to the rest of the predictor variables, GPs/MSOA was scaled to have mean 0 and 1 SD prior to model inclusion.

We additionally adjusted for the following variables derived from app response data, considered as percentage of responders within the MSOA: those who reported having kidney, heart or lung disease, and who are diabetic, a smoker or obese (calculated as BMI<30). We derived mean-adjusted age and sex variables to partially adjust for response bias (i.e. the extent responders in an MSOA represented the demographic of that MSOA). This was calculated as the difference of the expected mean/ratio of age/sex in the MSOA (derived from ONS population data) and the observed mean/ratio of age/sex amongst respondents.

We included a spatial lagged variable of the COVID-19 prevalence outcome. Inclusion of the lagged variable is one method that accounts for spatial autocorrelation (SAC)[4]. It attempts to adjust for spatial autocorrelation by capturing the variance explained by the influence of neighbouring regions on the value of interest – in this case COVID-19 severity/prevalence. The lagged variable is calculated at MSOA level by applying a spatial weights matrix (calculated in this instance under queen's

contiguity) to the outcome variable (in this case COVID-19 prevalence) and computing the lag using the function lag.listw in the 'spdep' R package. This variable is then included as a covariate within the model.

Data from eight time points were analysed , calculating the covariates (derived from app responders) and spatial lag at each time point, a dummy variable adjusting for the different sample times was included in the model as a random effect (allowing for a random intercept). MSOA was also included to allow for a random intercept to account for the repeat observations over the eight time periods, along with country as a fixed effect to account for difference in methodology in creation of IMD and RUC.

The users' distribution across GB is not uniform but all analyses took this into account by considering only middle super output areas (MSOAs) with at least 20 individuals reporting on the app (n = 8097, n removed = 387), and we included as a covariate the proportion of responders per MSOA at each time point, in order to adjust for differences in responders by MSOA. Analysis was conducted in RStudio v1.1.423 and R v3.6.3.

Variables were checked for multicollinearity before model inclusion using Spearman's correlation, (see **Figure B**) with the *a priori* threshold of > (+/-) 0.7 indicating a variable should be removed.

BMJ Publishing Group Limited (BMJ) disclaims all liability and responsibility arising from any reliance placed on this supplemental material which has been supplied by the author(s)

Figure B. Assessment of collinearity between the variables included in the MSOA-level mixed-effects models. Each cell of the matrix displays Spearman's correlation between two. The table is colour coded according to the Spearman's correlation, with blue denoting a positive correlation and red denoting a negative correlation. GP/MSOA= General Practitioners per middle super output area level; RUC= Rural-urban gradient; Av Household N= average household number.

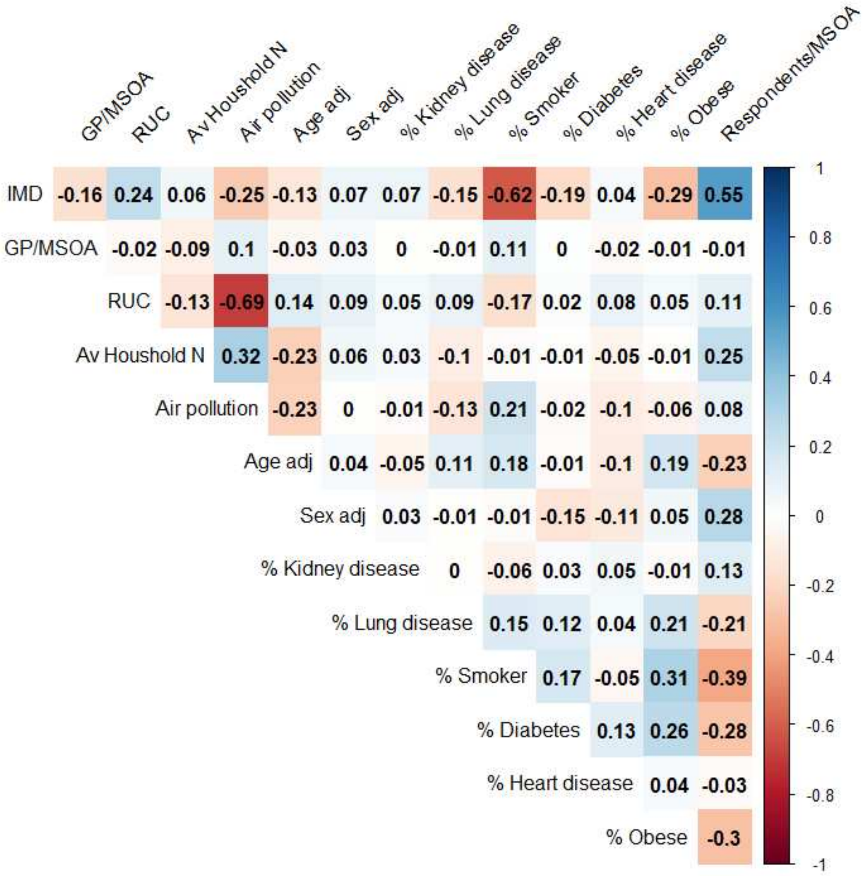

The model approach was therefore as follows:

- Model 1 (M1): Linear regression of the estimated COVID-19 prevalence and the IMD
- Model 2 (M2): Linear mixed effects model (LMM) of estimated COVID-19 prevalence and the IMD, adjusted for country, and allowed a random effect of MSOA ID and time (assuming random intercept for both)
- Model 3 (M3): Linear mixed effects model of estimated COVID-19 prevalence and the IMD, adjusted as above in M2, with additional adjustment for spatial autocorrelation (SAC) via inclusion of a spatial lag.

BMJ Publishing Group Limited (BMJ) disclaims all liability and responsibility arising from any reliance placed on this supplemental material which has been supplied by the author(s)

- Model 4 (M4): Linear mixed effects model as in M3, with the inclusion of geosocial mediators and confounders and proportion of MSOA population who were app users.
- Model 5 (M5): Linear mixed effects model as in M4, with the inclusion of aggregated co-morbidities as the % of respondents in MSOA with diabetes, kidney, lung or heart disease, who are obese or are smokers.
- Model 6 (M6): Covariate + mean-adjusted LMM – Linear mixed effects model as in M6, with the inclusion of mean-adjusted age and sex variables

Supplementary References

1. Menni C, Sudre CH, Steves CJ, et al. Quantifying additional COVID-19 symptoms will save lives. *Lancet* 2020;395(10241):e107-e08. doi: 10.1016/s0140-6736(20)31281-2 [published Online First: 2020/06/09]

2. Menni C, Valdes AM, Freidin MB, et al. Real-time tracking of self-reported symptoms to predict potential COVID-19. *Nat Med* 2020;26(7):1037-40. doi: 10.1038/s41591-020-0916-2 [published Online First: 2020/05/13]

3. Zhang C, Luo L, Xu W, et al. Use of local Moran's I and GIS to identify pollution hotspots of Pb in urban soils of Galway, Ireland. *Sci Total Environ* 2008;398(1-3):212-21. doi: 10.1016/j.scitotenv.2008.03.011 [published Online First: 2008/04/29]

4. Diniz-Filho JA, Nabout JC, de Campos Telles MP, et al. A review of techniques for spatial modeling in geographical, conservation and landscape genetics. *Genet Mol Biol* 2009;32(2):203-11. doi: 10.1590/S1415-47572009000200001 [published Online First: 2009/04/01]

Bowyer RCE, *et al. Thorax* 2021; 76:723–725. doi: 10.1136/thoraxjnl-2020-215119
