# Supplementary figures and images for: Geo-social gradients in predicted COVID-19 prevalence in Great Britain: results from 1 960 242 users of the COVID-19 Symptoms Study app
Source: Thorax. 2020 Dec 29;76(7):723–5. doi: 10.1136/thoraxjnl-2020-215119 (PMC8223682; doi:10.1136/thoraxjnl-2020-215119)

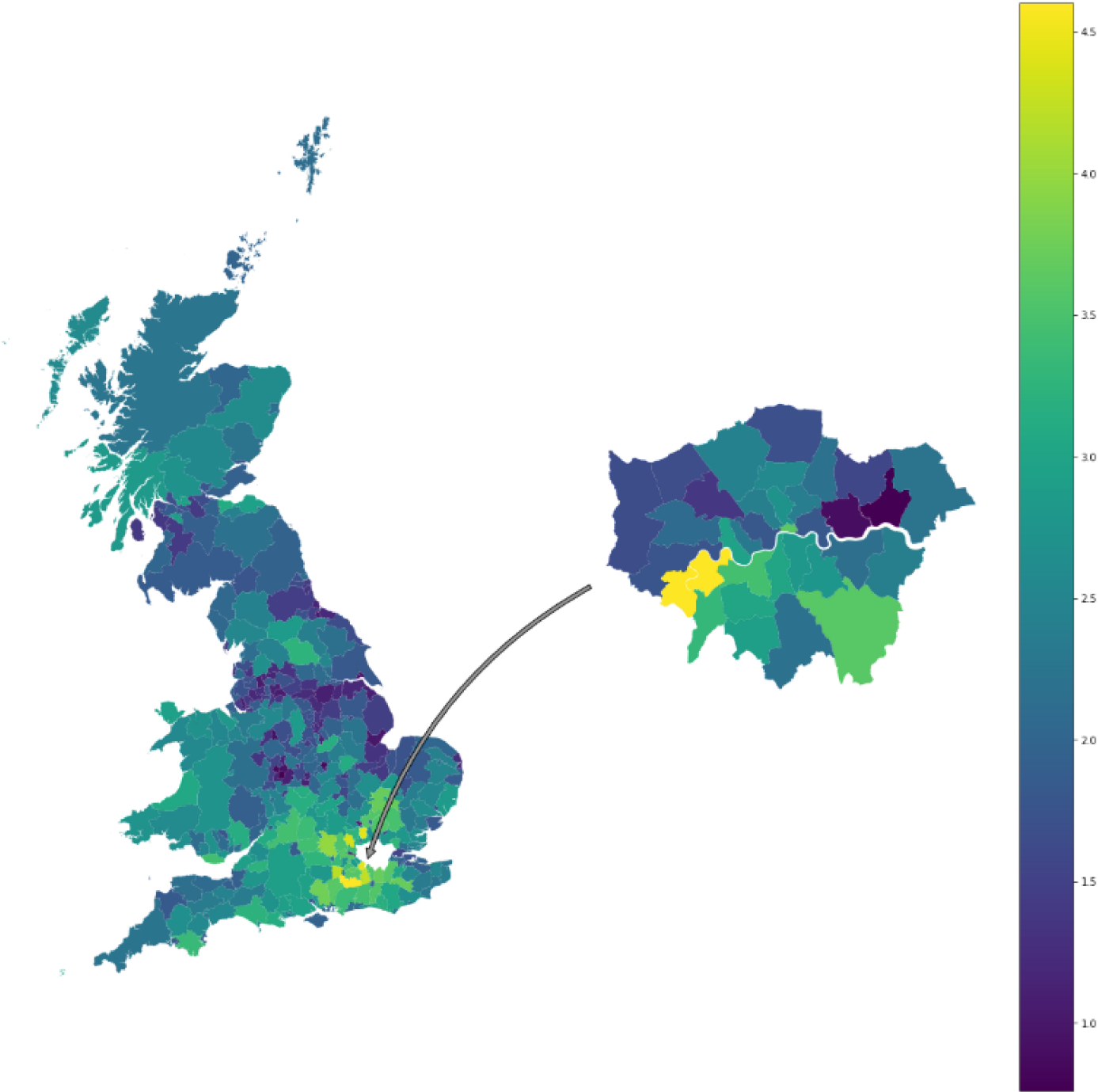

Supplement: Supplementary data [file thoraxjnl-2020-215119supp002.pdf]

### Predicted Covid-19 +ve cases in GB with highlighted spatially significant hotspots

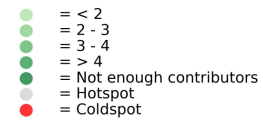

Supplement: Supplementary data [file thoraxjnl-2020-215119supp003.pdf]
